# Psychiatric disorders during acute hospital treatment of COVID-19 - a case series

**DOI:** 10.1192/j.eurpsy.2021.1736

**Published:** 2021-08-13

**Authors:** L.A. Fernandes, C. Ribeiro, M. Martins, J. Carreno, I. Guerra, C. Oliveira, C. Vieira, A. Luís, T. Maia

**Affiliations:** 1 Mental Health Department, Hospital Prof. Doutor Fernando Fonseca EPE, Amadora, Portugal; 2 Child And Adolescent Psychiatry Mental Health Department, Hospital de Dona Estefânia, Centro Hospitalar Universitário de Lisboa Central, Lisboa, Portugal; 3 Psychiatry Department, Hospital Distrital de Santarém, EPE, Santarém, Portugal; 4 Agrupamento De Centros De Saúde Sintra, Unidade de Saúde Familiar Monte da Luz, Queluz, Portugal; 5 Agrupamento De Centros De Saúde Sintra, Unidade de Saúde Familiar Mactamã, Queluz, Portugal

**Keywords:** delirium, liaison psychiatry, COVID-19

## Abstract

**Introduction:**

Coronavirus disease (COVID-19) has been associated with the development mental and behavioural symptoms and psychiatric disorders. This association is stronger in severe cases of the disease and in those needing inpatient treatment, particularly in intensive care units (ICU).

**Objectives:**

To determine the incidence of psychiatric disorders in a Portuguese hospital-based sample of patients with COVID-19. To describe relevant demographic and clinical data.

**Methods:**

We reviewed all COVID-19 inpatients assessed by liaison psychiatry at our hospital between April and September 2020. Patients admitted due to a psychiatric disorder were excluded from the analysis. We reviewed medical records and retrieved relevant clinical data. ICD-10 was used to classify diagnoses.

**Results:**

We identified 36 cases with a mean age of 62.64 years-old (SD 19.23). The most common disorder was delirium, which occurred in 41.7% of our sample (15 patients), followed by adjustment disorder (22.2%, n=8), and depressive episode (16.7%, n=8). Most patients had no personal (61.1%, n=22) nor family (75%, n=27) history of a psychiatric disorder. Mean length of admission was 36.89 days (SD 28.91). Seventeen cases (47.22%) had at least one risk factor for severe COVID-19 disease and 14 (38.89%) were admitted at some point to the ICU.

**Conclusions:**

In our sample, delirium was the main cause for mental or behavioural symptoms in COVID-19 patients. However, we observed a wide array of presentations in our center. A larger sample would allow to better characterize this often-overlooked symptoms and identify risk factors to psychiatric syndromes.

**Disclosure:**

No significant relationships.

